# How case reports have contributed to the progress of surgery

**DOI:** 10.1186/s40792-014-0002-4

**Published:** 2015-01-16

**Authors:** Norihiro Kokudo

**Affiliations:** Hepato-biliary-pancreatic Surgery Division, Artificial Organ and Transplantation Division, Department of Surgery, Graduate School of Medicine, The University of Tokyo, 7-3-1 Hongo, Bunkyo-ku, Tokyo 113-8655 Japan

## Editorial

The now well-known Zollinger-Ellison syndrome was recognized by Professor Zollinger at Ohio State University and his student, Dr. Ellison, based on their insightful observations of two young women who suffered from refractory peptic ulcers requiring repeated surgery [1]. The index case for Kasabach-Merritt syndrome was a 2-month-old male infant who underwent radiation therapy for a huge and growing capillary hemangioma in the left thigh causing extensive purpura [2]. Dr. Kasabach was a radiologist and Dr. Merritt was a colleague pediatrician at Columbia University. The first successful right lobectomy of the liver was performed by Professor Honjo at Kyoto University in 1949 to remove a large liver metastasis from rectal cancer in a 22-year-old man [3]. Professor Honjo reported this epoch-making progress in liver surgery as a case report in an English journal. The first three cases of human liver transplantation were reported by Professor Starzl from the University of Colorado in 1963 [4]. Nevertheless, case reports or case series are ranked as low level 4 evidence [5] in the hierarchy of evidence-based medicine. Most of the top medical or surgical journals now restrict the acceptance of case reports to an extreme degree because such reports, in general, are seldom cited and thus would reduce the journal's impact factor. However, we must recognize how previous reports of a few unusual cases, observed and treated by excellent physicians, have contributed greatly to the progress of medical science.

In addition to the outstanding case reports mentioned above, there have been a number of amazing discoveries made by surgeons in their daily practice. Sir James Cantlie, a surgeon at the University of Hong Kong, started his anatomical research on the liver after he encountered an autopsy case of a remarkably shrunken right liver and a hypertrophic left liver arising from a liver abscess [6]. Based on this observation, he proposed the concept of the liver mid-plane, otherwise well known as the Rex-Cantlie line. In 1982, the first clinical application of preoperative portal vein embolization (PVE) was performed by Dr. Makuuchi at the National Cancer Center, Tokyo [7]. The concept of PVE occurred to him after he had encountered patients undergoing major hepatectomy for hilar cholangiocarcinoma. He noted that their right portal vein was occluded by the tumor invasion and their contralateral left liver was already hypertrophic before the operation, allowing for an uneventful postoperative course without any signs of liver failure.

The Japan Surgical Society has always encouraged young surgeons to submit interesting or educational case reports. Now, we have launched a new case report journal named *Surgical Case Reports*, a sister journal to compliment *Surgery Today*. We welcome surgeons worldwide to submit case reports to our new journal, which will be a completely open-access online journal. I anticipate that our new journal will contribute significantly to the progress of surgery.
